# Orientational Packing of a Confined Discotic Mesogen in the Columnar Phase

**DOI:** 10.3390/ijms11030943

**Published:** 2010-03-08

**Authors:** Wenjun Zheng, Ya-Ting Hu, Cheng-Yan Chiang, Chi Wi Ong

**Affiliations:** 1 Department of Photonics, National Sun Yat-Sen University, 70 Lienhai Road, Kaohsiung 80424, Taiwan; E-Mails: m953050046@student.nsysu.edu.tw (Y-T.H.); d963050006@student.nsysu.edu.tw (C-Y.C.); 2 Department of Chemistry, National Sun Yat-Sen University, 70 Lienhai Road, Kaohsiung 80424, Taiwan; E-Mail: cong@mail.nsysu.edu.tw (C.W.O.)

**Keywords:** discotic liquid crystal, columnar phase, self-assembly, hexaazatriphenylene

## Abstract

The stacking of discotic molecules (hexakis(alkoxy)diquinoxalino[2,3-a:2′,3′-c]phenazines) in the columnar phase sandwiched between two flat glass substrates has been studied. The surface free energy of the substrates, measured by means of sessile drop technique, is found to have significant influence on the way that the discotic molecules anchor on the surface, and a steady thermal state of the system is crucial for a homogenous orientation of the discotic columns. On a surface of high free energy, the discotic molecules anchor with their disc-face toward the surface. A decrease in the surface free energy of the substrate causes the discotic columns to tilt away from the normal of the substrate.

## 1. Introduction

Discotic liquid crystals (DLCs), which consist of disc-like molecules and exhibit columnar phases, have attracted more and more attention due to their potential in electrooptic and photonic applications. In the columnar phases, the discotic molecules spontaneously aggregate along a unique direction and assemble in a one-dimensional molecular stack, which can further organize into higher ordered two-dimensional lattices. Proper control of the thermal state of a discotic mesogen during molecular stacking allows the maintenance of the molecular ordering in columnar phases and the attainment of ordered glassy states [1]. In the oriented columnar stack of discotic molecules, there is a preferential direction for charge carrier transportation, and thus a well aligned DLC exhibits highly mono-dimensional carrier mobility along the column direction [2]. As a result, DLCs are thought to be particularly useful for applications in which an efficient unidirectional charge transport is desired, and have been found to have uses in light emitting diodes [3], photovoltaic cells [4,5], and organic semiconductor transistors [6,7]. It is crucial to control molecular stacking of DLCs to form a highly ordered columnar phase for achieving a good device performance, and thus a good knowledge on the mechanisms behind the molecular stacking of DLCs is of primary importance.

**Figure 1.**
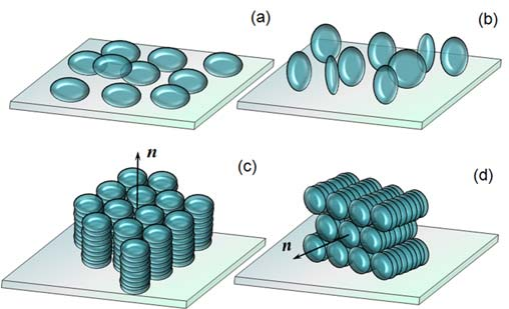
(a) Face-on anchoring of discotic molecules. (b) Edge-on anchoring of discotic molecules. (c) Homeotropic alignment of discotic columns. (d) Homogenous alignment of discotic columns.

The molecular axis of a discotic molecule is generally defined as the symmetric axis normal to the disc surface. Similarly to the mesogens that consist of rod-like molecules, the director of the DLC is a unit vector, which is the local average orientation of the axes of the discotic molecules, and the ordering of the DLCs is described by the features of director field. In general, discotic molecules tend to stack face-to-face through a self-organization due to the *π*–*π* interaction, and form a columnar structure [8]. In an assembly of discotic molecules, which are put in contact with a solid surface, two basic types of molecular anchoring of the discotic columns on the substrate are usually found: the face-on and the edge-on. In the face-on anchoring, the discotic molecules lie on the substrate with the axes of molecules perpendicular to the substrate surface, whereas in the edge-on anchoring, the molecular axes are parallel to the surface of the substrate. In a columnar phase, the molecular packing in which all discotic molecules are in the face-on anchoring and stack to form discotic columns organized with their axes orienting perpendicular to the substrate is referred to as the homeotropic alignment of the columnar phase. The homogeneous alignment of the columnar phase is defined as the molecular packing in which all molecules are in edge-on anchoring and organize in the columns oriented with their axes parallel to the substrate surface (Figure 1). Recently, we conducted research to study the aggregation of discotic molecules sandwiched between two solid substrates, with the aim to find out the conditions for an orientational molecular stack of DLCs in a confined space.

On a solid surface, the way that the disc-like molecules anchor to the surface and the orientation of the discotic columns are determined by many factors, such as geometrical shape of molecules, intra-and intermolecular interaction, surface topography of substrate, molecule-solid surface interaction, etc. The surface energetic state of the substrate has been shown to be a crucial factor that decides the type of the discotic molecule anchoring and the orientation of the discotic columns [9], and has attracted more and more attention [10]. These indicate that the nature of the surface on which a discogen assembles has huge impact on the molecular stacking of discotic molecules. When a DLC is put on a solid substrate without cover, a planar alignment of the columnar phase is usually obtained as a result of the balance between the DLC-solid and the DLC-air surface tensions, respectively [11]. Moreover, the self-organization of the discotic molecules is usually bound to a thermal process. For thermotropic compounds, molecular aggregation normally occurs during transfer from a higher symmetrical state to a lower one when the temperature drops. The features of the molecular aggregation in a columnar phase will be strongly affected by the thermal conditions, such as temperature fluctuation in a domain and temperature gradient between neighboring regions, *etc*. The examination of the effects of thermal conditions on the molecular stacking in the columnar mesophase is one of the objectives of the present studies reported in this article.

Knowledge of orientation of the discotic columns is critical for understanding the mechanism of the molecular stacking and determination of the type of alignment of the columnar phase. We examined the orientation of the discotic columns by means of orthoscopic observation using a polarizing optical microscope (POM). When all molecules in a layer of DLC sandwiched between two substrates are aligned to orient in a unique direction, the DLC layer acts like a wave plate with optical axis orienting parallel to the director of the DLC layer. When the aligned DLC layer is sandwiched between two crossed polarizers, the intensity of the transmitted light is given by the equation

(1) $$I=I_{o}{\text{sin}}^{2}(2\phi ){\text{sin}}^{2}\left(\frac{\pi R}{\lambda }\right)$$

where *φ* is the azimuthal angle between the optical axis of the DLC and the optical axis of the polarizer of POM, *R* = *Δn*·*d* is the retardation, *d* is the thickness of the DLC layer, *Δn* = (*n*_e_ – *n*_o_) is birefringence of the DLC layer, *n*_e_ and *n*_o_ are refractive indices for extraordinary and ordinary rays, respectively.

In this article, we demonstrate that the surface free energy of the substrates is of primary importance in determining the type of the alignment of the columnar phase. The thermal state of discotic compound will strongly influence the progress of the molecular stacking and is crucial for an orientational assembly of discotic columns.

## 2. Results and Discussion

**Figure 2.**
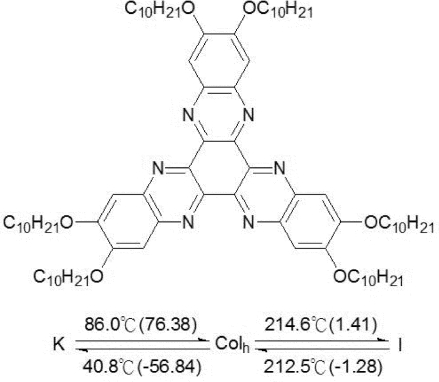
Chemical structure and phase sequence of 2,3,8,9,14,15-hexakis(decyloxy)-diquinoxalino-[2,3-a:2′3′-c]phenazine (LC10).

The discotic mesogen used in the present studies was synthesized in house and labeled LC10. The label is used in the following discussion for convenience. As illustrated in Figure 2, the core of the discogen consists of diquinoxalino[2,3-a:2′,3′-c]phenazine (DQP), also called hexaazatrinaphtalene (HATN); attached to it are six alkoxy side chains. This structure favors aromatic stacking and leads to the formation of a hexagonal columnar mesophase. LC10 exhibits a columnar phase in a broad temperature region although the solid→columnar phase transition temperature is quite high (86.0 °C). The HATN core is found to maintain the electron-deficient characteristic, and the incorporation of alkoxy chains does not override the electron deficiency of the HATN core. The HATN-based mesogen shows high carrier mobility, and is expected to be used as an acceptor.

**Figure 3.**
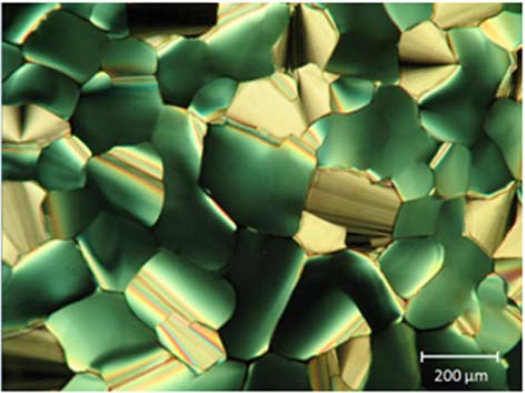
Optical microtexture of a layer LC10 which was cooled down at a rate of 10 ºC/min. The photograph was taken when the sample was at 120 °C.

The discotic molecules were allowed to stack in a confined space determined by two glass substrates. The thickness of the DLC layer has not been optimized for the device performance, and was arbitrarily fixed at 10 μm for all samples under investigation in the present study. A sandwiched LC10 layer was heated to 230 °C, which is well above the clearing temperature of the discogen, and then allowed to cool down at a rate of 10 °C/min. The molecular stacking occurred during the cooling course. Figure 3 shows a photomicrograph of the LC10 layer at a temperature of 120 °C, where the mesogen is in the columnar phase. The mosaic-like texture of the mesogen indicates the formation of the columnar domains in the LC layer. Although there are a few domains in which the fan shape texture indicates the packed molecules are in the edge-on anchoring, the majority of domains exhibit a quite uniform and murky texture. When the sample was rotated in the POM, the optical transmission over the entire sample was small and nearly unchanged; however, a completely dark state of the domains could not be achieved. A small variation in the brightness of those domains with uniform texture was observed as the sample rotated in the POM. The small light transmission of the domains indicates that the discotic molecules were in the face-on anchoring on the surface of the substrate in the domains. The periodic variation in light transmission for each single domain suggests that the molecules are oriented in one direction and the director of the domain tilts a small angle away from the normal to the substrate, and a non-synchronism in light transmission from one domain to another suggests that the directors of the domains orient in different directions. The tilt of the director will be analyzed in more detail in a following section.

**Figure 4.**
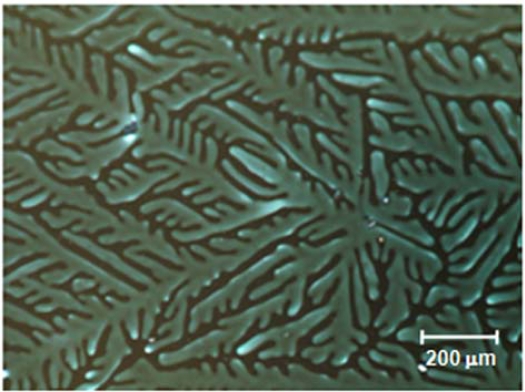
LC10 in a confined cell made using glass plates. The sample was cooled down to 120 °C from an elevated temperature at a cooling rate of 0.1 °C/min. The pattern reflects a hexagonal symmetry of the columnar structure with the face-on molecular anchoring.

Better molecular stacking, in terms of orientational order of the columns, can be achieved when the discogen is slowly cooled down. It was found that the cooling rate of the discogen must not exceed 0.3 °C/min to obtain a good orientational columnar stacking. Figure 4 shows a photomicrograph of a LC10 layer that was cooled down at a rate of 0.1 °C/min. The optical texture of the sample reflects a typical hexagonal symmetry of the columnar phase [12]. This, together with the face-on molecular anchoring, which has been confirmed by the optical examination of the sample, allows us to conclude that the DLC layer is in homeotropic alignment.

The experimental results reveal the importance of a steady thermal process for an ordered stacking of discotic molecules. Brown *et al*. [8] showed that for non-covalent bonding self-assembly of the disc-like molecules in a discotic compound, the long-range Van der Waals forces and the *π*-*π* coupling of the neighbouring benzene rings are responsible for the self-organization and the order in discotic compounds. For most disc-like molecules, the *π*-*π* interaction produces the preferential face-to-face stacking between molecules. During the molecular stacking, the discotic molecules adjust their positions relative to one another. The orientational ordering of the molecules in the first monolayer at the surface is duplicated and epitaxially grows into bulk through self-assembly. During molecular stacking, a fast changing in the temperature of the discogen due to a quick cooling process will produce temperature gradient between adjacent regions resulting in the creation of local turbulence that may disturb the face-face coupling of discotic molecules leading to breaking or destroying the orientated molecular stacking. Therefore, to achieve an oriented stacking, the assembly of the molecules must progress steadily.

The hexagonal columnar assembly of the discotic molecules in the mesophase can be attributed to the large and flat aromatic core in molecular structure of LC10. In a confined space, the way that the discotic molecules anchor on the substrate and the aggregation of the discotic columns in the cell are hugely governed by the driving forces created due to the surface coupling between the substrate and the discotic molecules. Therefore, the surface characteristics of the substrate are believed to be of primary importance in determining the orientation of the discotic columns. Herein, we consider the influence of the surface free energy of the substrates on the orientation of the discotic columns.

**Figure 5.**
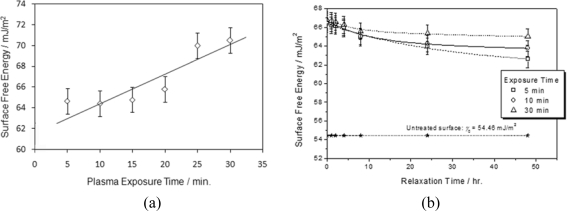
Surface free energy of the substrate versus the exposure period of the substrate to plasma.

The surface free energy of glass substrates was modified by O_2_ plasma buffing. Cleaned glass substrates possess a surface free energy of 54.46 mJ/m^2^. The O_2_ plasma processing caused a marked increase in the surface free energy of the glass substrates. The surface free energy of the substrates was found to increase with the duration of the exposure of the surface to the plasma (Figure 5a). The increase in the surface free energy was thought to result from an increase in the free radicals on the glass surface and the electrostatic interaction arising from electron collision with surface and electrification during the plasma bombardment [13,14]. Besides, the increase in oxygen on the surface due to the extra amount of oxygen brought to the surface by the O_2_ plasma stream [15–19] may also contribute to the increase in the surface free energy. In a clean room environment, the surface energetic state of O_2_ plasma can be held for quite a long time. After the treated substrates were left in a Class 4 clean room for one working day (eight hours), their surface free energies dropped a small fraction, less than 3% (Figure 5b), and the longer they were exposed to O_2_ plasma, the longer the substrate kept its surface at a high energy level. A buffing direction on the substrates was created by obliquely bombarding the surface with the plasma stream [20]. In the experiment, the substrates in the chamber were tilted by an angle of 15°. The buffing direction is parallel to the project of the direction of the incident plasma stream on the substrate. It has been shown that the anti-parallel cell configuration, in which the plasma buffing direction on each of both substrates orients in an opposite direction, is of requisite for achieving an aligned DLC layer [20]. All cells used in the present study were constructed in anti-parallel configuration.

**Figure 6.**
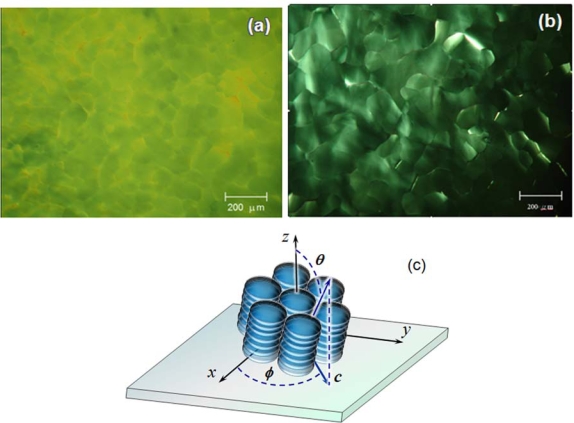
Photomicrographs of the LC10 layers in the cells with their substrates buffed by O_2_ plasma for (a) 5 min and (b) 15 min. (c) Molecular tilt of homeotropically aligned DLC. Tilt angle *θ* is defined as the angle between the director ***n*** of the DLC and the normal to the surface, *i.e.*, the z axis. The azimuthal angle *φ* is defined as the angle between the project of ***n*** on the surface and the plasma buffing direction, which is parallel to the x axis.

**Figure 7.**
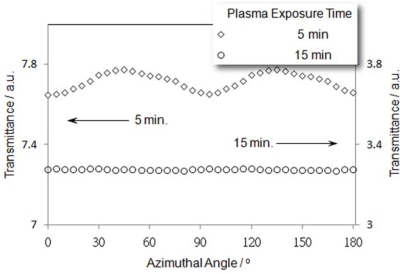
Light transmittance of LC10 layers sandwiched by substrates that were O_2_ plasma bombarded for different period. Prolongation of the exposure period of the substrates to the plasma can cause a decrease in light transmittance of the DLC samples.

Samples were prepared by sandwiching LC10 in the cells whose substrates were processed by exposure to O_2_ plasma for different durations. In these samples, no evidence that supports the chemical reaction has taken place at DLC/substrate interface has been found. To keep the surface free energies of the substrates used for subsequent experiments to be consistent with the measured data, all cells were constructed within two hours after the completion of the plasma treatment of the substrates, and DLC samples were prepared shortly after the cells were constructed. All samples exhibited a very low light transmission, which can be attributed to small phase retardation (cf. Equation 1) of the DLC layers due to a vertical alignment of the discotic columns [20]. Figure 6 shows photomicrographs of two LC10 layers in cells whose substrates were buffed with O_2_ plasma for 5 min and 15 min, named as M5 and M15, respectively. Both DLC layers exhibit a rather uniform optical texture, and a quite small light transmittance. The uniform optical texture of the samples indicates that the molecules in both layers orient in a unique direction, *i.e.*, they are aligned. When the M5 sample was rotated in the POM, its appearance varied bright and dark alternately although the magnitude of the variation was small. This phenomenon can be reflected by the response of the optical transmittance of the sample to the variation in the azimuthal angle (Figure 7). A prolongation in the duration of the plasma exposure causes the surface energy of the substrates to increase, and leads to changes in columnar alignment on the substrate and the DLC layer, as can be seen from M15 (Figure 6b), which exhibited a darker appearance. The intensity of transmitted light from the sample, as can be seen from Figure 7, is small and will not vary with rotating the sample. The optical behavior of the two samples shows a typical effect caused by an induced birefringence created due to a deviation of propagation direction of light from the optical axis of the DLC medium. If the director of a homeotropically aligned columnar phase DLC layer tilts an angle *θ* away from the normal to the substrate, for a normally incident light ray, the effect refractive index *n*_eff_ can be given as

(2) $$n_{eff}=\frac{n_{e}n_{o}}{\sqrt{{\left(n_{e}\text{cos}\theta \right)}^{2}+{\left(n_{o}\text{sin}\theta \right)}^{2}}}$$

If *θ* is very small, sin*θ* ~ 0, then *n*_eff_ ~ *n*_o_/cos*θ*. Substituting *n*_eff_ into Equation (1) yields

(3) $$I^{\prime }=I_{o}{\text{sin}}^{2}(2\phi ){\text{sin}}^{2}\left[\frac{\pi d}{\lambda }\left(\frac{1-\text{cos}\theta }{\text{cos}\theta }\right)n_{o}\right]={I^{\prime }}_{o}{\text{sin}}^{2}(2\phi )$$

If the director of the homeotropic alignment columnar phase, *i.e.*, the axis of the columns, makes a small angle *θ* against the normal to the substrate, (1-cos*θ*)/cos*θ* can be very small, thus *I*_o_′ becomes small. As *I*_o_′ does not vanish, so a sinusoidal variation in transmitted light intensity with small amplitude can be observed when the azimuthal angle *φ* varying, and this is what we have seen in M5 sample. Therefore, the LC10 layer in M5 is in homeotropic alignment with the columns tilt a small angle from the normal to the substrate.

For the sample M15, a lower light transmittance (Figure 7), and an azimuthally unchanged light transmittance of the sample can be attributed to a vanishing birefringence of the DLC layer with all other parameters in Equation (1) remaining unchanged. This can happen when the columns in the sample are in homeotropic alignment, *i.e.*, *θ* = 0. In this case, Equation (2) becomes *n_eff_* = *n_o_*, and Equation (3) yields *I* → 0. Although we can only provide here a qualitative analysis due to a lack in the knowledge of *n_o_* and *n_e_* for LC10, the observed results indicate that between substrates with higher surface energy, the discotic columns will tend to arrange in a homeotropic alignment.

**Figure 8.**
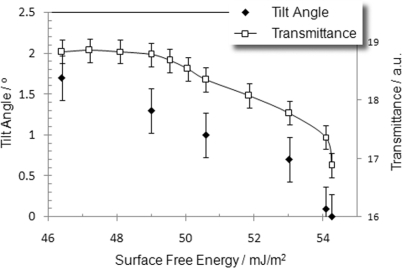
Effect of surface free energy of the substrate on molecular orientation and light transmittance of the DLC layers.

As shown in a previous section, the prolongation of the plasma bombardment can increase the surface free energy of the substrate. The changes in the energetic state of the surface may cause a change in the way the discotic molecules anchor and/or the orientation of discotic columns on the surface. It is extremely difficult to measure pretilt angle for columnar phase without the knowledge of the birefringence of the compound. However, to estimate pretilt angle for a homeotropic aligned columnar phase can be very simple. We estimated the angle that the discotic columns tilted away from the normal to the substrate by means of crystal rotation method [21]. The correlation between the tilt of the discotic columns and surface free energy is shown in Figure 8. As can be seen, the tilt of discotic columns of the DLC sandwiched between the substrates decreases with an increase in the surface free energy due to a prolongation of the period of O_2_ plasma bombardment. As a summary, the following conclusion, based on the experimental observation, can be drawn. A substrate possessing a high surface free energy supports a face-on molecular anchoring for DLC; if the change in the temperature of the DLC is slow and steady, the face-on molecules can stack to form columns with their axes parallel to each other, *i.e.*, in the homeotropic alignment with a unique director. When the surface free energy of the substrate is low, the discotic columns will tilt from the normal to the layer. The tilt angle increases with the decrease in the surface free energy, and eventually, the edge-on anchoring of the discotic molecules at the surface is achieved when the surface free energy of the substrate, as reported previously [9], is sufficiently low.

## 3. Experimental Section

The discotic compound used in the present study is 2,3,8,9,14,15-hexakis(decyloxy)-diquinoxalino-[2,3-a:2′3′-c]phenazine (a derivative of HATN). The details on the synthesis of the compounds are given elsewhere [22,23]. Phase transition temperatures of the compound were determined by means of differential scanning calorimetry and optical microscopy. The chemical structure and the phase sequences of the compound are shown in Figure 2. The compound exhibits a hexagonal columnar mesophase, which has been confirmed by the X-ray diffraction (XRD) measurements [23], in a wide temperature range, and a quite significant supercooling behavior at Col/Crystal transition. It has been revealed by XRD measurement that in the columnar phase molecules of LC10 tend to organized into hexagonal structure with a 26.8Å core-core distance [23].

**Figure 9.**
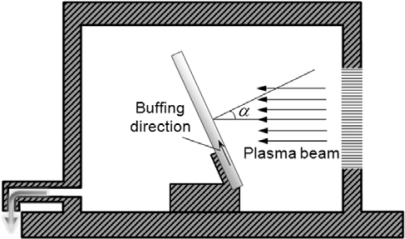
Schematic showing the chamber for plasma surface treatment. The sample is installed with the normal to the surface tilting an angle *α* away from the plasma beam. The pressure of the chamber is maintained at 1.6 mbar.

Substrates used in the present study were flat glass slides. The surfaces of the substrates were treated by O_2_ plasma buffing. Plasma treatment of the substrates was carried out using a microwave plasma system (PS 400, PVA TePla). The chamber of the plasma generator is schematically shown in Figure 9. A stream of a mixture of oxygen and argon was used to produce plasma. The fluxes of oxygen and argon were controlled at 800 ml/min and 80 ml/min, respectively. The chamber pressure was maintained at 1.6 mbar. Ultrasonically cleaned substrates were put into the chamber, and were set to tilt from the incident direction of plasma beam. We defined the angle between the direction of the incident plasma beam and the normal to the substrate surface as incident angle *α* of the plasma beam (Figure 9). The project of incident direction of the plasma beam on the substrate is referred to the plasma buffing direction.

The surface energy of the substrates after O_2_ plasma treatment was determined by means of sessile drop measurement using a DSA100 surface tension meter (KRÜSS, Germany). For this purpose, contact angles between standard reference liquids and relevant substrates were measured, and the substrates were characterized for surface energy by application of the Owens-Wendt model [24].

For the sandwiched samples, cells were made using two plasma treated substrates. The thickness of the cells is controlled at 10 μm using proper spacers. Samples were prepared by putting a small amount of the discotic compound on the opening of the cell. The compound was forced into the cell by capillary action at an elevated temperature. The temperatures of the samples were controlled using a Linkam TMS93 temperature system (Linkam Scientific Instrument Ltd). The alignment of the samples was examined by means of orthoscopic observation that was performed using a polarizing microscope (Axioscop 40, ZEISS). Optical measurement was carried out using a photodiode detector that was mounted in the microscope. Photomicrographs were taken using a digital camera (PowerShot A620, Canon) that is installed in the microscope. A halogen lamp installed in the polarizing microscope was used as the light source.

## 4. Conclusions

We have studied the molecular stacking of a hexaazatriphenylene based discotic mesogen LC10 in a confined space, and examined the effects of surface free energy of the substrates and thermal states of the DLC on the molecular stacking. We modified the energetic state of the substrates by means of O_2_ plasma buffing. The O_2_ plasma treatment causes an increase in the surface free energy of the substrate, and improves the hydrophilicity of the glass surface. The surface free energy of the substrate is a decisive factor that will govern the way the molecular anchoring of the discotic molecules. On a surface that possesses a higher free energy, the molecular aggregation with the face-on anchoring of discotic molecules is usually achieved, whereas a surface that possesses low free energy will favor the edge-on anchoring. The thermal state of the mesogen during molecular stacking was found to be crucial for an orientational columnar aggregation. For LC10, to produce an oriented columnar stacking, the decrease in sample temperature must be controlled at a speed below 0.3°C/min. A fast change in temperature may result in the occurrence of turbulence leading to breaking or destroying the ordered molecular stacking.
